# The efficacy of ^99m^Tc-HYNIC-PSMA SPECT/CT in detecting primary lesions and metastasis in newly diagnosed prostate cancer

**DOI:** 10.3389/fonc.2023.1165694

**Published:** 2023-06-02

**Authors:** Taisong Wang, Lingzhou Zhao, Wenli Qiao, Na Sun, Jinhua Zhao, Yan Xing

**Affiliations:** Department of Nuclear Medicine, Shanghai General Hospital, Shanghai Jiao Tong University School of Medicine, Shanghai, China

**Keywords:** prostate cancer, primary tumor, metastasis, 99mTc-HYNIC-PSMA, SPECT/CT

## Abstract

**Purpose:**

Compared with PET/CT or PET/MRI, SPECT/CT is cheaper and more readily accessible. This study was designed to investigate the efficacy of ^99m^Tc-HYNIC-PSMA SPECT/CT in detecting primary tumors and metastases in patients with newly diagnosed prostate cancer (PCa).

**Methods:**

A retrospective analysis of 31 patients with pathologically proven PCa was performed at Shanghai General Hospital from November 2020 to November 2021. Planar whole-body imaging was performed on all patients with a SPECT/CT scan of PSMA-positive regions 3–4 h after intravenous injection of 740 MBq ^99m^Tc-HYNIC-PSMA. Positive PSMA uptake lesions were evaluated, and SUVmean and SUVmax were measured in each lesion. Associations between SPECT/CT parameters and clinicopathologic factors (tPSA and Gleason Score) were analyzed. The diagnostic capability of SPECT/CT parameters, tPSA, and GS in distant metastatic detection was evaluated by logistic regression.

**Results:**

The SUVmean and SUVmax of the high-risk stratification subgroups (tPSA>20 ng/ml, GS ≥8, and tPSA >20 ng/ml and GS≥8) were higher than those of the low-moderate risk stratification subgroups, with sensitivities of 92% and 92%, respectively. Neither SPECT/CT parameters (SUVmean, SUVmax) nor clinicopathologic factors (tPSA, GS) had high sensitivity (80%, 90%, 80%, and 90%, respectively, P <0.05) in distant metastatic prediction. For both the guideline tPSA level (20 ng/ml) and the cut-off level (84.3 ng/ml), the difference in the distant metastasis detection rate between the low predicted tPSA group and the high predicted tPSA group was statistically significant (0% *vs*. 47.62%, *P* = 0.005; 9.09% *vs*. 88.89%, *P* = 0.000, respectively). Twenty patients with pathological 99mTc-PSMA avid only in the prostate beds underwent radical prostatectomy. Seven of them underwent lymph node dissection, a total of 35 lymph nodes were removed, and no lymph nodes were detected with metastasis, which was consistent with ^99m^Tc-HYNIC-PSMA SPECT/CT.

**Conclusion:**

^99m^Tc-HYNIC-PSMA SPECT/CT is effective in the risk stratification and distant metastasis detection of primary PCa patients. It is of great value in guiding treatment strategies.

## Introduction

The global incidence and disease burden of prostate cancer (PCa) are still increasing annually, which remains a great challenge due to limited medical resources (1). Prostate cancer is the fourth most common cancer in the world (7.3% of all cancers) and the fifth leading cause of cancer death in men (6.8%) (2). Prostate cancer is highly heterogeneous, which directly affects prognosis and treatment decisions (3). Early-stage PCa can be treated with surgery and anti-androgen endocrine therapy, and the 5-year survival rate is close to 100%, which is expected to achieve the purpose of radical cure; however, the five-year survival rate for metastatic PCa is only 36%–54%, and the median survival time is approximately 42 months (4). Based on the American Urological Association (AUA) and the European Association of Urology (EAU) guidelines, high-risk prostate cancer patients are defined as having a total prostate-specific antigen (tPSA) level >20 ng/ml and/or a Gleason score ≥8. The probability of distant metastasis and mortality in high-risk patients is significantly increased, which is not suitable for radical resection of prostate cancer and radiotherapy (5–7). However, previous studies have found that predicting the presence or absence of distant metastases based solely on tPSA levels is not reliable and may lead to many unnecessary prostate biopsies (7, 8). In these cases, it is necessary to develop an objective and accurate imaging method as a risk stratification tool for prostate cancer.

Prostate-specific membrane antigen (PSMA) is a transmembrane protein specifically expressed in prostate cancer and tumor neovasculature. PSMA expression was correlated with the malignancy and aggressiveness of PCa tumors (9, 10). Previous studies have shown that 68Ga-PSMA PET/CT imaging has a high primary tumor detection rate and can provide accurate staging of lymph node metastasis in patients with PCa (11). The degree of PSMA uptake in primary prostate cancer was correlated with the tPSA level and GS before treatment (12). Furthermore, the most commonly used semiquantitative indicator of PET/CT is the maximum standardized uptake value (SUVmax), and positron nuclide-labeled PSMA inhibitors have been used to evaluate primary and metastatic lesions in patients with medium-high-risk prostate cancer (13, 14). However, positron nuclides are expensive, so ^99m^Tc-labeled small-molecule PSMA inhibitors are attracting more attention. In recent years, a novel ^99m^Tc-labeled PSMA inhibitor (^99m^Tc-HYNIC-PSMA) has been synthesized and shown good affinity for tumors (15, 16). The synthesis process of ^99m^Tc-HYNIC-PSMA is very simple and quick, which is more convenient for clinical applications. Compared with PET/CT, SPECT/CT is more widely distributed around the world, and the imaging agent is cheaper, which is more promising for the application of PSMA.

This study was conducted to retrospectively investigate the role of ^99m^Tc-HYNIC-PSMA SPECT/CT semiquantitative parameters, tPSA levels, and Gleason score in newly diagnosed PCa patients.

## Materials and methods

### Patients

**Figure 1:**
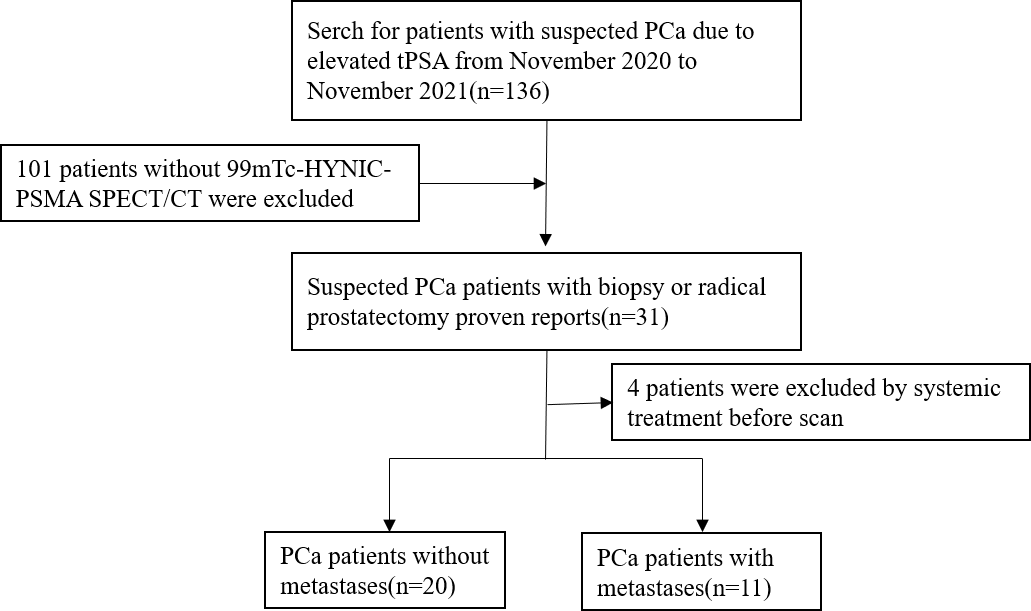
The flowchart of patient enrollment.

Ethical approval was obtained from the Clinical Ethics Committee of Shanghai General Hospital (No. 2018-15). Informed consent was obtained from all individual participants included in the study. Thirty-one patients with prostate cancer diagnosed by biopsy or radical prostatectomy were included in this retrospective study from November 2020 to November 2021. All patients underwent ^99m^Tc-HYNIC-PSMA SPECT/CT, and serum PSA was detected within one month prior to imaging. Patient exclusion criteria were as follows: 1) local or systemic treatment; and 2) unavailable imaging data. The flowchart of patient enrollment is provided in **Figure 1**.

### Radiosynthesis and quality control of 99mTc-HYNIC-PSMA

HYNIC-PSMA was manufactured by Hangzhou ThinHeal Pharma-Tech Co., Ltd. (Hangzhou, China). ^99m^Tc-pertechnetate solution (Na99mTcO4) was purchased from Shanghai Atomic Kexing Pharmaceutical Co. Ltd. (Shanghai, China). Tricine, ethylenediamine-N,N’-diacetic acid (EDDA), stannous chloride (SnCl2), acetonitrile (CH3CN), trifluoroacetic acid (TFA), and phosphate-buffered saline (PBS) were purchased from Shanghai Macklin Biochemical Co., Ltd. (Shanghai, China). Other chemicals and solvents were supplied by Sinopharm Chemical Reagent Co. Ltd. (Shanghai, China).

^99m^Tc-HYNIC-PSMA was prepared following the procedure reported in the literature (15, 17). In brief, 10 μl of HYNIC-PSMA solution in water, 500 μl of EDDA solution (20 mg/ml in 0.1 M NaOH), 500 μl of tricine solution (40 mg/ml in 0.2 M PBS), and 50 μl of SnCl2 solution (1 mg/ml in 0.1 M HCl) were mixed. After the addition of 1,000 μl of Na99mTcO4 solution (30–120 mCi/ml), the reaction mixture was heated for 20 min at 100°C and allowed to cool to room temperature.

The final solution was filter-sterilized using a 0.22 μm Millipore needle filter, and a bacterial endotoxin test was performed using an Endosafe portable test system. The radiochemical purity (RCP) of ^99m^Tc-HYNIC-PSMA was analyzed using an Agilent 1260 high-performance liquid chromatography system equipped with a UV‐vis detector (λ = 220 nm) and a radioactive flow detector. A SunFire C18 column (5 μm, 4.6 × 250 mm) was used at a 1 ml/min flow rate with the following solvent system: 0.1% TFA in H2O and CH3CN (0–20 min, 10%–20% CH3CN).

### ^99m^Tc-HYNIC-PSMA SPECT/CT acquisition

All ^99m^Tc-HYNIC-PSMA SPECT/CT imaging was performed on a single device (Discovery NM/CT 670, General Electric Medical Systems, Waukesha, WI) after IV administration of approximately 740 MBq (20 mCi) of ^99m^Tc-PSMA, a low-energy all-purpose collimator. Three to four hours after injection, whole-body images were acquired in the supine position with a table speed of 10 cm/min, a matrix size of 256 × 256, and an energy window width of 140 keV. SPECT imaging parameters: 30 s/frame, total 60 frames; matrix size 128 × 128, zoom 1.0. The CT image parameters were 120 kV and 90 mAs. The iterative method (four iterations, eight subsets) was used for image reconstruction. Xeleris software (General Electric) was applied to review planar and fusion SPECT/CT images.

### Image analysis

**Figure 2:**
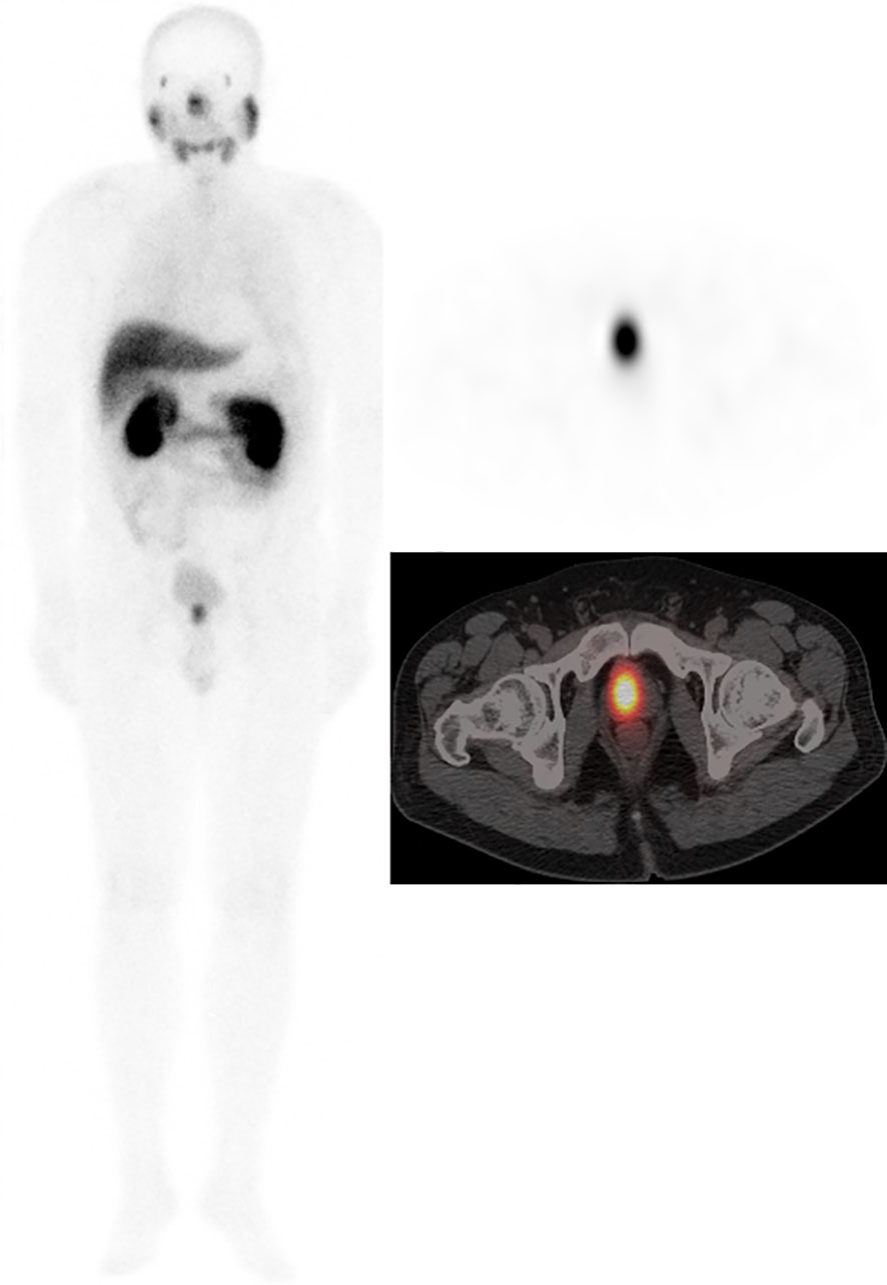
Male, 62 years, GS = 7 (3 + 4). tPSA 62.65 ng/ml. ^99m^Tc-HYNIC-PSMA SPECT/CT imaging showed primary prostate cancer on whole-body scan planar (ANT) imaging (left). Axial SPECT image (upper right) and axial fused image (bottom right) showed the primary tumor in the right prostate lobe.

**Figure 3:**
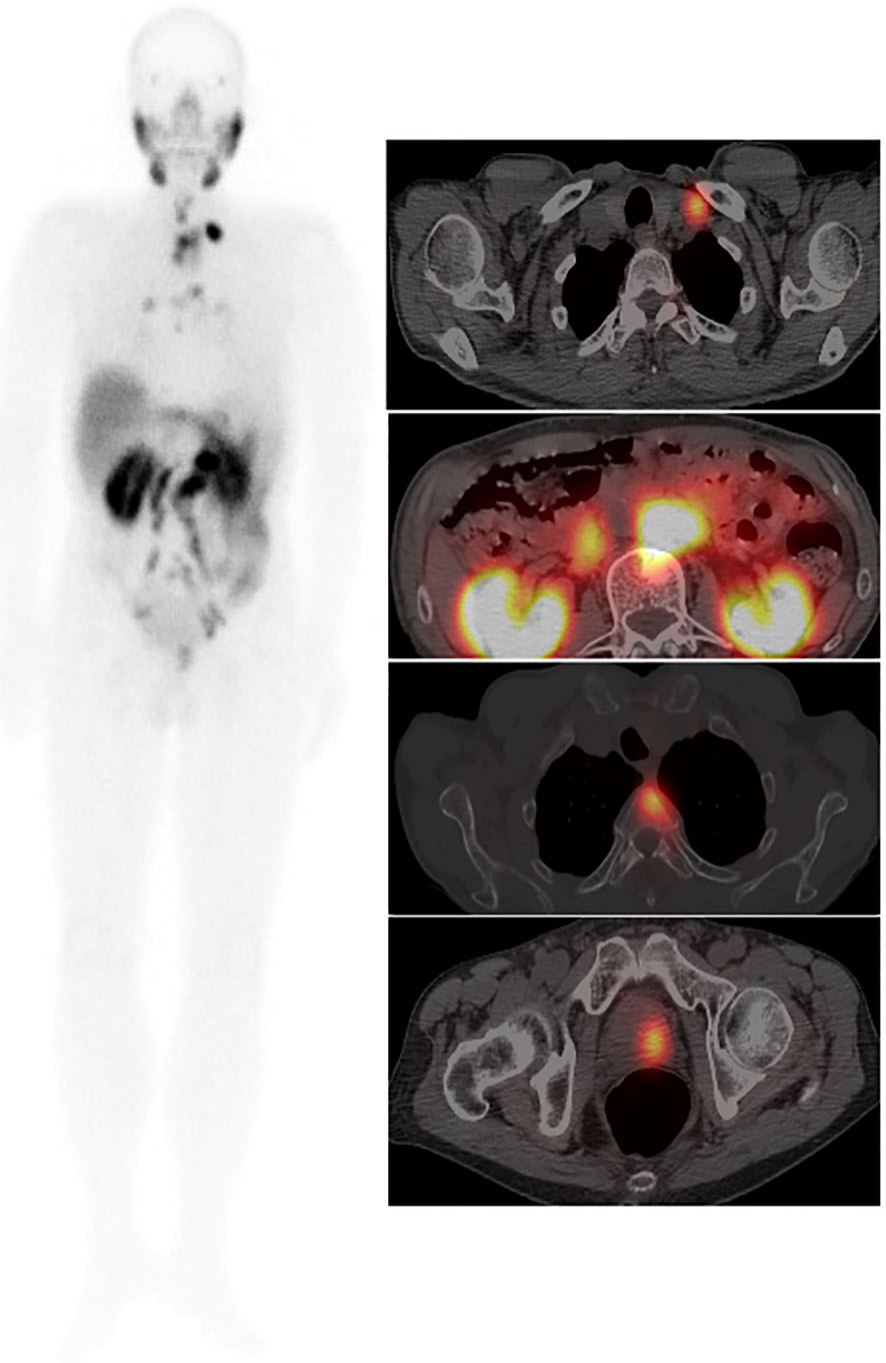
Male, 76 years, GS = 8 (4 + 4). tPSA 147.0 ng/ml. ^99m^Tc-HYNIC-PSMA whole-body scan planar (ANT) image (left) showed multiple PCa lesions. An axial fused image (right) showed primary tumors in the left prostate lobe, multiple lymph node metastases, and thoracic vertebral metastases.

Two nuclear medicine physicians with more than 10 years of experience in reading SPECT/CT images jointly reviewed SPECT/CT images. When the two physicians disagreed, a consensus was reached through discussion. According to the drug metabolism characteristics of PSMA, the nasopharynx, salivary gland, liver, spleen, intestine, kidneys, and bladder may have different degrees of physiological distribution on SPECT/CT images. After excluding physiological or obvious nonprostate cancer-related uptake, the foci where ^99m^Tc-HYNIC-PSMA uptake was higher than that of the surrounding normal tissue were defined as positive. The primary lesions and metastases of each tissue or organ were counted (**Figures 2**, **3**).

After inputting the patient’s height, weight, and injection parameters, the SPECT parameters (SUVmax and SUVmean) of the primary tumor were measured automatically with a manually adapted isocontour threshold centered on lesions with focally increased radioactive tracer uptake corresponding to the tumor site verified by prostatic needle biopsy. The SPECT parameter values for metastasis lesions were also calculated at the same time.

### Standard reference for imaging results

Owing to ethical and practical reasons, not all lesions can receive pathology results. All available information, including pathology, ^99m^Tc-MDP bone scans, CT, MRI, and clinical data, was used as a standard reference.

### Statistical analysis

Statistical analysis was performed using dedicated software (SPSS 25.0; IBM Corp., Armonk, NY). Numerical data are presented as medians, means, standard deviation ranges, or percentages. Pearson correlation was used to analyze the correlation of tPSA level and Gleason score with SPECT/CT indexes. Subgroup analyses were performed using the Wilcoxon–Mann–Whitney U test. The ability of SPECT/CT parameters and clinicopathological factors to predict risk stratification and distant metastasis was analyzed by ROC curve and logistic regression. *P* <0.05 was the significance level (two-tailed).

## Results

The RCP of ^99m^Tc-HYNIC-PSMA was calculated to be greater than 99% by radio-HPLC, and the endotoxin test showed a negative result. Details of the participants are summarized in **Table 1**. A total of 287 lesions were identified by ^99m^Tc-HYNIC-PSMASPECT/CT, including 31 primary prostate tumors and 256 metastatic lesions. One patient had regional lymph node metastasis; 10 patients had distant metastasis, and twenty patients had no metastasis. The SPECT semiquantitative parameters (SUVmax and SUVmean) of the tPSA ≤20 ng/ml, Gleason Score <8, and low-intermediate risk groups were higher than those of the tPSA >20 ng/ml, Gleason Score ≥8, and high-risk groups. The details are shown in **Table 2**. SUVmean and SUVmax were significantly related to Gleason Score, tPSA, and risk stratification (rs = 0.456, 0.439, 0.424, rs = 0.468, 0.474, and 0.450, respectively, P <0.05).

**Table 1:** Details of 31 participants.

Characteristic	Value
**age**	68(65, 88)
Median (range)	71(54-88)
Mean±SD	69.8±7.9
tPSA level (ng/mL)	
Median (range)	37.9(2.14-147)
Mean±SD	62.2±54.6
**≤20**	10(32.3%)
**>20**	21(67.7%)
Gleason Score	
G6	1(3.2%)
G7	10(32.3%)
G8	5(16.1%)
G9	12(38.7%)
G10	3(9.7%)
Risk Group	
Low-intermediate risk	6(19.4%)
High risk	25(80.6%)
**Non-metastatic Patients (%)**	20(64.5%)
**Metastatic Patients (%)**	11(35.5%)
Localization of PSMA positive lesions	
Prostate region	31
Lymph nodes	85
Bone	170
Liver	1
**SUVmax of prostate region**	21.9±21.4
**SUVmax of metastases**	34.1±18.0

**Table 2:** ^99m^Tc-HYNIC-PSMA SPECT/CT parameters of different subgroups.

Categorical Variable	PSA≤20	PSA>20	Sig	GS<8	GS≥8	Sig	Low-intermediate risk(n=6)	High-risk (n=25)	Sig
SUVmean	8.66±5.44	12.37±7.08	*P*=0.001	10.55±11.65	14.02±14.25	*P*=0.000	5.17±1.15	7.53±1.53	*P*=0.000
SUVmax	23.18±16.08	32.58±21.69	*P*=0.002	23.52±15.16	33.67±20.48	*P*=0.000	21.69±15.19	30.51±20.47	*P*=0.000

**Figure 4:**
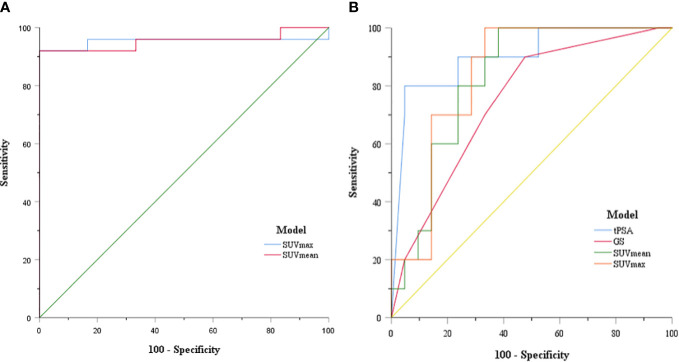
**(A)** ROC curve of SUVmax and SUVmean for PCa patient risk stratification; **(B)** ROC curve of SUVmax, SUVmean, tPSA, and GS for prostate cancer distant metastasis risk prediction.

The ability of SUVmean and SUVmax to predict risk stratification was evaluated by receiver operating characteristic (ROC) curve analysis. The area under the ROC curve (AUC) of SUVmean was 0.953 (95% CI: 0.880–1.000, *P* = 0.001). The AUC of SUVmax was 0.953 (95% CI: 0.874–1.000, *P* = 0.001). The sensitivity and specificity for the SUVmean were 92% and 100%, respectively (cut-off value 6.80). The sensitivity and specificity for the SUVmax were 92% and 100%, respectively (cut-off value 10.66) (**Figure 4A**). SUVmax (SUVmax of primary tumor), SUVmean (SUVmean of primary tumor), tPSA, and Gleason score in predicting the risk of distant metastasis were analyzed by ROC curve analysis simultaneously. The details are shown in **Table 3** (**Figure 4B**).

**Table 3:** The results of SUVmean, SUVmax, tPSA, and Gleason score in predicting PCa metastasis.

parameter	AUC	*P*	95% CI	Sensitivity	Specificity	Cut-off
SUVmean	0.824	0.004	0.680~0.968	80%	76.2%	19.0
SUVmax	0.838	0.003	0.698~0.978	90%	71.4%	23.1
tPSA	0.902	0.000	0.783~1.000	80%	95.2%	84.3
GS	0.748	0.028	0.569~.926	90%	52.4%	8

**Figure 5:**
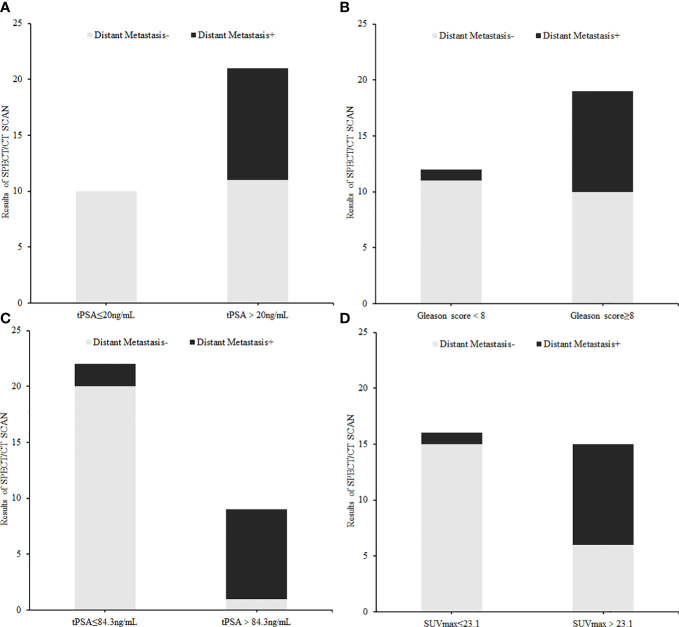
The difference in distant metastasis findings on ^99m^Tc-HYNIC-PSMA SPECT/CT between each index subgroup: **(A)** tPSA subgroup (≤20 ng/ml *vs*. >20 ng/ml), **(B)** Gleason Score group (<8 *vs*. ≥8), **(C)** Cut off tPSA subgroup (≤84.3 ng/ml *vs*. >84.3 ng/ml), **(D)** Cut off SUVmax subgroup (≤23.1 *vs*. >23.1).

The correlation between tPSA level, Gleason Score, and distant metastasis shown on ^99m^Tc-HYNIC-PSMA SPECT/CT was analyzed. Compared with the tPSA ≤20 ng/ml group, the detection rate of distant metastasis was higher in the tPSA >20 ng/ml group (0% *vs*. 47.62%, respectively; P = 0.009; **Figure 5A**). A similar result prevailed in the Gleason score <8 group and ≥8 group (8.33% *vs*. 47.37%, respectively; P = 0.026; **Figure 5B**). Based on the tPSA ROC curve analysis results, all patients were divided into two prediction groups (tPSA ≤84.3 ng/ml and >84.3 ng/ml). The distant metastasis detection rate was higher in the tPSA >84.3 ng/ml group than in the tPSA ≤84.3 ng/ml group (9.09% *vs*. 88.89%, respectively; P = 0.000; **Figure 5C**). The high prediction SUVmax (>23.1) group also had a higher detection rate than the low prediction SUVmax (≤23.1) group for distant metastasis (6.25% *vs*. 60%, respectively; P = 0.002; **Figure 5D**).

The performance of the tPSA level and Gleason score to predict metastasis shown on ^99m^Tc-HYNIC-PSMA SPECT/CT was further analyzed. The tPSA and GS group methods recommended in the guide were selected as independent variables, and then a logistic regression was performed. The actual metastasis results were input into the analytical procedure as a binary variable. The results of logistic regression indicated that high (≥8) and low (<8) Gleason scores were independent predictors of metastasis (OR = 12.222, 95% CI: 1.306–114.399, P = 0.028).

Twenty patients without metastatic ^99m^Tc-PSMA avid underwent radical prostatectomy. Seven of 20 patients underwent lymph node dissection; a total of 35 lymph nodes were removed, and no lymph nodes were positive for PCa by pathology. After 3–16 months of follow-up, biochemical recurrence occurred in only one patient, and the PSA level was 0.29 ng/ml. Eleven patients with distant metastatic ^99m^Tc-PSMA avid were given palliative treatment, and the original treatment strategy was changed.

## Discussion

In this study, the clinical value of ^99m^Tc-HYNIC-PSMA SPECT/CT for risk stratification differentiation and distant metastasis prediction in primary PCa was evaluated. The study showed that the ^99m^Tc-HYNIC-PSMA SPECT/CT indexes SUVmax and SUVmean can contribute to risk stratification and distant metastasis risk prediction, which is consistent with a recent 18F-PSMA-1007 PET/CT study (14). Logistics regression analysis reflected that patients with a high Gleason Score (≥8) gained more benefits from ^99m^Tc-HYNIC-PSMA SPECT/CT, which can be used as a screening index for patients undergoing ^99m^Tc-HYNIC-PSMA SPECT/CT.

It is generally thought that 68Ga-PSMA PET/CT imaging is superior to ^99m^Tc-PSMA SPECT/CT imaging in detecting primary lesions due to its superior resolution. However, in the study of García-Pérez et al., it was found that due to the long half-life of ^99m^Tc, hydration delay imaging can be performed and a better target-to-background ratio image can be obtained. Therefore, more delayed pelvic imaging may eliminate the limitation of ^99m^Tc-PSMA SPECT/CT imaging in displaying the primary focus, which needs to be confirmed by more research data in the future (18). In this study, the primary tumor detection rate was 100% (31/31), showing that the performance of ^99m^Tc-PSMA SPECT/CT imaging in primary tumor detection is remarkable as well.

In the detection of metastases, 68G-PSMA PET/CT imaging has high sensitivity, which leads to the transformation of patients from a non-metastatic disease state detected by traditional imaging to a metastatic disease state, thus changing the treatment strategy (19). Using 68Ga-PSMA-11 PET/CT, Fendler found that 55% of M0 castration-resistant prostate cancer patients had metastatic lesions that were not identified by conventional imaging (20). Klabunde et al. compared the ability of ^99m^Tc-PSMA imaging and ^99m^Tc-MDP whole-body bone imaging to detect bone metastases and found that the former was superior to the latter in terms of staging, restaging, and monitoring of biochemical recurrence after radical prostatectomy. It can also discover extraosseous metastases, which are expected to replace ^99m^Tc-MDP whole-body bone scintigraphy and help identify candidates for PSMA-based internal radiation therapy (21). In this group of patients, 11 patients had metastases. In addition to lymph node metastases, five patients had bone metastases, and one had liver metastases. These patients all changed their treatment strategies to avoid an unnecessary radical prostatectomy.

For prostate cancer patients undergoing radical resection, whether to perform pelvic lymph node dissection and the extent of dissection have been topics of long-term discussion in the urinary community. From a diagnostic standpoint, pelvic lymph node dissection is the gold standard for lymph node staging. However, with the introduction of PSA screening, the lymph node metastasis rate has dropped from 40% to 20% in newly diagnosed patients, which makes pelvic lymph node dissection exempt for most patients and reduces postoperative complications. Therefore, assessing which patients can be exempted from lymph node dissection is particularly important. A recent meta-analysis summarized the value of PSMA PET/CT imaging in guiding lymph node dissection in prostate cancer patients and found that PSMA PET/CT imaging had a high NPV (97%) based on lymph node analysis, and PSMA PET/CT scan had a high NPV (97%), which provided promising accuracy in the field of lymph node staging for PCa. The high NPV in men with a lower risk of LNI might be clinically useful to reduce the number of unnecessary pelvic lymph node dissection (PLND) procedures performed. However, in high-risk patients, negative PSMA PET/CT cannot replace staging an enlarged PLND (22). In this study, ^99m^Tc-PSMA SPECT/CT imaging was perfectly consistent with pelvic lymph node dissection results, which showed a good ability to predict lymph node metastasis.

Our study also has certain limitations, such as the small number of enrolled patients. It must be noted that the PSMA molecular probe used in this study is still excreted by the urinary system, which may lead to a reduced signal of metastases around the bladder and kidneys, potentially obscuring the visibility of tumor lesions. We cannot completely rule out the influence of the results of this study.

## Conclusions

In summary, ^99m^Tc-HYNIC-PSMA SPECT/CT is effective in the risk stratification and distant metastasis detection of primary PCa patients. It is of great value in guiding treatment strategies. ^99m^Tc-HYNIC-PSMA SPECT/CT is suitable for popularization and application in underdeveloped areas.

## Data availability statement

The original contributions presented in the study are included in the article/supplementary material. Further inquiries can be directed to the corresponding authors.

## Ethics statement

The studies involving human participants were reviewed and approved by The Clinical Ethics Committee of Shanghai General Hospital. The patients/participants provided their written informed consent to participate in this study.

## Author contributions

TW drafted the manuscript and contributed to the data collection, examination, and analysis. LZ contributed to the preparation of materials and acquisition of data. WQ and NS contributed to the interpretation of data. JZ and YX contributed to the conception of the study and revision of the manuscript. All authors contributed to the article and approved the submitted version.
